# Contact and complexity in English varieties: The influence of speaker numbers on syntheticity and grammaticity

**DOI:** 10.1371/journal.pone.0341167

**Published:** 2026-01-27

**Authors:** Katharina Ehret

**Affiliations:** Department of English, University of Freiburg, Freiburg, Germany; Father Muller Charitable Institutions, INDIA

## Abstract

Empirical research on language complexity has shown that languages and varieties can and do differ in their complexity. One of the key triggers responsible for this observed variation is language contact as non-native acquisition. The influence of language contact on complexity is, however, not uncontroversial: While a number of large-scale typological studies have reported that language contact decreases complexity, others find no such effect in their data. This paper offers a corpus-based perspective on the influence of language contact on morphosyntactic complexity in an English-varieties context. Precisely, I model the effect of the number of native speakers, the proportion of non-native speakers and language type–a theoretical construct based on the sociolinguistic contact history of the varieties–in a corpus database of 25 spoken English varieties. Morphosyntactic complexity is here operationalised as the number of bound grammatical markers (syntheticity) and the total number of explicit grammatical markers (grammaticity). The models show that the number of native speakers negatively correlates with syntheticity. However, contrary to theoretical expectations, the proportion of non-native speakers shows a weak positive effect on syntheticity. None of the speaker-related triggers influences grammaticity. Only language type shows a consistent negative effect on both syntheticity and grammaticity indicating that historic language contact scenarios do impact complexity. The crucial question, then, is what (non-)native speaker numbers really represent and if they are a (good) proxy for language contact. Overall, the results corroborate the controversial findings in the typological literature highlighting the importance of how complexity is operationalised.

## Introduction

This paper is situated at the intersection of corpus-based variationist linguistics and sociolinguistic typology and contributes to research on language complexity. Language complexity has been a widely discussed and also hotly debated topic for about two decades now [1–3]. Language complexity is also a truly multi-faceted concept and no uniform definition or measure exists (for an overview of different definitions see [4]). However, two broad distinctions are generally made. First, local and global complexity are distinguished [5]. Global complexity refers to the overall complexity of a language. In contrast, local complexity refers to complexity at various linguistic levels, such as morphology, syntax, or phonology. In fact, the bulk of complexity research focuses on local complexity. Second, existing complexity measures are categorised into absolute, system-inherent measures and relative, user-focused measures such as learning difficulty [5]. This paper takes an absolute approach to complexity and draws on local, morphosyntactic measures.

Originally centring around the question of whether all languages were, on the whole, equally complex [6], research on language complexity has increasingly focused on how to best measure complexity [7] and evaluate existing metrics [8,9]. In the meantime, language complexity has become a measurable and well-researched aspect of variation. In this context, a substantial body of empirical studies has shown that languages and varieties can and do differ in their complexity at local linguistic levels [3,10,11]. The major question currently under debate in the theoretical linguistics community is the extent to which socio-demographic triggers are responsible for this observed variation. In particular, language contact is one of the key triggers frequently discussed. Common proxies for language contact in the literature are the proportion of non-native speakers [12,13] and the number of native speakers [12,14,15].

The idea of language contact as determinant of language complexity has been around for a while. According to the sociolinguistic typology outlined in [16,17], three types of contact situations, which favour either complexification or simplification, can be distinguished. Low-contact situations in which languages are spoken by isolated, small, and closely-knit speech communities favour the maintenance or increase of complexity. In contrast, high-contact situations involving high rates of adult second language (L2) acquisition favour a decrease in complexity [16]. Finally, high-contact situations of intensive and long-term contact, often involving large rates of child bilingualism, favour an increase in complexity [16,18]. [15] have codified this idea in the Linguistic Niche Hypothesis distinguishing between esoteric (low-contact) and exoteric (high-contact with L2 acquisition) speaker communities. The influence of language contact on complexity is, however, not uncontroversial. Although a number of empirical large-scale typological studies provide evidence for the fact that language contact decreases complexity [13,19], other, more recent studies provide contrary evidence for the effect of language contact on complexity [20,21].

Against this background, I model the influence of language contact as non-native acquisition on the morphosyntactic complexity in a corpus database of 25 typologically diverse, spontaneous spoken English varieties. Language contact is operationalised as the proportion of non-native speakers and the number of native speakers. The theoretical construct language type is also included in the models as a proxy for historic language contact. Morphosyntactic complexity here is implemented as syntheticity and grammaticity [22]. Syntheticity measures the number of bound grammatical markers and grammaticity measures the total number of explicit grammatical markers in a corpus text. In this vein, I tap the International Corpus of English for indigenous second language and high-contact first language (L1) varieties around the world, the Freiburg Corpus of English Dialects for low-contact and high-contact L1 varieties in the British Isles, and the Santa Barbara Corpus of Spoken American English. Methodologically, state-of-the-art regression modelling is utilised. Based on recent empirical evidence [19,23] and the contact scenarios described in the literature [16,18], the working hypothesis is that higher numbers of native speakers and larger proportions of non-native speakers lead to a decrease in morphosyntactic complexity.

However, this hypothesis is only partially supported by the data. The results show that the number of native speakers is negatively correlated with syntheticity, whereas the proportion of non-native speakers is positively associated with syntheticity. Neither of the speaker-related triggers shows an effect on grammaticity. Instead, here geography is the best predictor. Language type also consistently shows a negative effect on morphosyntactic complexity. Overall, these results mirror the empirical controversy regarding the influence of language contact reported in the typological literature. Precisely, they highlight that different morphosyntactic features vary in the degree to which they respond to language contact. Furthermore, the paper provides usage-based evidence, i.e. evidence based on naturalistically produced language data, to a debate which is largely based on atlas evidence. The paper also makes an important theoretical contribution as it raises the fundamental question of what (non-)native speaker numbers really represent and if they are a (good) proxy for language contact.

## Materials and methods

This section describes the corpus database, the extra-linguistic triggers, and the statistical analyses. All numeric data, detailed statistical outputs, and other supplementary materials discussed in this paper are available at https://github.com/Morphosyntactic-Variation-in-Englishes/syngram-complexity. Demographic materials are available at https://github.com/Morphosyntactic-Variation-in-Englishes/DOVE.

### Corpus database

To furnish a case study, a typological corpus database of spontaneous spoken English varieties was compiled that matches the range of different variety types in the electronic World Atlas of Varieties of English (eWAVE, [24]). eWAVE is the largest available database for English varieties sampling a total of 77 spontaneous spoken English varieties. In this spirit, I tap the International Corpus of English (ICE, https://www.ice-corpora.uzh.ch/en.html), the Freiburg Corpus of English Dialects (FRED, https://freidok.uni-freiburg.de/proj/1), and the Santa Barbara Corpus of Spoken American English (SBCSAE, https://www.linguistics.ucsb.edu/research/santa-barbara-corpus, [25]). In addition to the varieties listed in eWAVE, three other ICE varieties (Canadian English, British English, and Trinidadian English) and two FRED varieties (English dialects in the Midlands, Hebridean English) were added to increase the number of observations and the representativeness of the sample.

ICE is a synchronic corpus collection of comparative corpora and samples national varieties of English around the world. Individual corpora typically comprise one million words of running text across several written (e.g., letters, newspaper) and spoken registers (e.g., face-to-face conversation, broadcast). However, this analysis is restricted to spoken face-to-face conversation, i.e. texts labelled as S1A, because the interest here is in spontaneous spoken English. ICE corpora typically sample rather “educated” English [26], i.e. English which is close to (some variety of) standard English. In contrast, FRED is the most extensive corpus of traditional spoken varieties (*aka* dialects) in the British Isles. FRED is based on oral history interviews from nine major British dialect areas and contains roughly 2.5 million words. The SBCSAE comprises 249,000 words of spoken interactions which primarily consist of face-to-face conversations but also include other spoken language use (e.g. card games, story-telling, or a university lecture). The SBCSAE is thus a natural match for the texts sampled in S1A in ICE.

Although the three corpora all sample naturalistic, spontaneous spoken language, they are not directly comparable in terms of register, speaker type, and speaker age. Nevertheless, for the current purposes, i.e. the analysis of typologically diverse English varieties, the materials are all considered sufficiently close to spontaneous spoken conversation. Moreover, the three corpora provide an adequately representative sample of different English language types. ICE and the SBCSAE are representative of high-contact L1 and indigenous L2 varieties around the world, whereas FRED is representative of high-contact and traditional low-contact L1 varieties. Finally, differences between the three corpora are statistically controlled for.

Part of FRED and some of the ICE corpora are fully part-of-speech (POS) annotated with the C7 tag set (http://ucrel.lancs.ac.uk/claws7tags.html), a tag set specifically for English. Thus, if POS-annotated versions of the corpora were available, these annotated versions were used. Otherwise, the texts were automatically POS-annotated with the CLAWS tagger [27] using the C7 tag set (http://ucrel.lancaster.ac.uk/claws/).

In total, the database comprises ten high-contact L1 varieties (e.g., Irish English, Singapore English), nine indigenous L2 varieties (e.g., Indian English, Ugandan English), and six low-contact varieties (e.g., Scottish English, English dialects in the Midlands) (see Table 1). The classification of English varieties into different language types is adopted from eWAVE [24] and is informed by the dialectological and sociolinguistic literature [16,17]. Although the aim was to create a typologically and geographically balanced as well as representative sample, corpus availability restricted the selection of varieties, especially in regard to low-contact varieties. The total database covers 25 varieties and comprises 1927 text files.

**Table 1:** Overview of English varieties by type, number of texts, and corpus.

Variety	Type	Texts	Corpus
Australian English	high-contact L1	100	ICE-AU**
British English	high-contact L1	100	ICE-GB
Canadian English	high-contact L1	100	ICE-CAN
American English	high-contact L1	60	SBCSAE**
Singapore English	high-contact L1	100	ICE-SIN
Ghanaian English	indigenous L2	101	ICE-GH
Hebridean English	traditional L1	41	FRED-Heb**
Hong Kong English	indigenous L2	100	ICE-HK
Indian English	indigenous L2	100	ICE-IND
Irish English	high-contact L1	100	ICE-IRE
Jamaican English	indigenous L2	100	ICE-JA
Kenyan English	indigenous L2	27	ICE-EA**
Manx English	high-contact L1	2	FRED-Man**
Midlands dialects	traditional L1	58	FRED-Mid
New Zealand English	high-contact L1	100	ICE-NZ
Nigerian English	indigenous L2	67	ICE-NIG**
North of England dialects	traditional L1	61	FRED-North*
Philippine English	high-contact L1	100	ICE-PHI
Scottish English	traditional L1	55	FRED-ScL* & ScH**
Southeast of England dialects	traditional L1	59	FRED-SE*
Southwest of England dialects	traditional L1	85	FRED-SW*
Sri Lankan English	indigenous L2	100	ICE-SL**
Trinidadian English	indigenous L2	100	ICE-TT**
Ugandan English	indigenous L2	100	ICE-UG**
Welsh English	high-contact L1	11	FRED-Wal**
Total		**1927**	

Corpora which were partially annotated within the project are marked with * and fully annotated within the project with **.

### Extra-linguistic triggers

The extra-linguistic triggers analysed as main predictors are the two demographic triggers number of native speakers and proportion of non-native speakers in a given country/territory. These predictors are common proxies for language contact in the typological literature [11,14,15]. The analyses furthermore include the non-numeric predictor language type and model the geographic predictor region as a random effect.

All extra-linguistic information is taken from A socio-demographic Dataset fOr Varieties of English v1.0 (DOVE, [28]), which was specifically compiled to investigate the relationship between language complexity and extralinguistic triggers in English varieties. DOVE draws on numerous open-access sources, mainly census data or (non-)governmental surveys but also builds on information from eWAVE [24]. It includes, among a number of other extra-linguistic triggers, data on native and non-native speakers. As most speaker data in DOVE was extrapolated from censuses or similar surveys, the number of native speakers refers to speakers who have reported speaking a language as their “mother tongue”, “native language”, “main language”, or “home language”. The number of non-native speakers refers to speakers who have acquired English as a second language or as one of their additional languages, and who have reported some degree of proficiency in English. Sometimes, speaker numbers are approximated via some other variable, e.g. educational attainment (for details see the documentation in [28]). Note that the number of native and non-native speakers comprises only the number of speakers in a given country or territory in which the variety is primarily spoken thus excluding, e.g., diaspora speakers [28]. In this paper, the terms native and non-native speaker are adopted as is customary in the sociolinguistic-typological complexity literature. On a conceptual plane, non-native speakers are defined as adult second language learners, following [12] and [14]. Thus, it is assumed that large-scale (adult) second language acquisition and the accommodation of adult second language learners by native speakers lead to the simplification of morphosyntax as suggested by iterated learning experiments [29,30] and psycholinguistic experimental set-ups [31]. The proportion of non-native speakers is calculated as the proportion of non-native speakers in the total speaker population, whereby the total population comprises both native and non-native speakers [14]. The number of native speakers is also often used to approximate language contact as non-native acquisition. The idea is that languages spoken by larger native speaker communities are also frequently acquired and spoken outside these communities [15]. Although speaker numbers are frequently used in the typological literature, their appropriateness as a proxy for language contact is rarely discussed. In fact, it is unclear whether present-day speaker numbers adequately reflect the historic population numbers and make-up which gave rise to present-day languages and varieties (for a discussion of time depth see also [14]).

Language type is a theoretical construct which is based on the socio-historical background, including the historic language contact situation, of individual English varieties [24]. Language type is included in the analyses for two reasons. Firstly, previous research suggests connections between morphosyntactic complexity and language type of English varieties [32,33]. Secondly, the focus in this paper is on language contact, and language type conveniently approximates historic language contact. The English varieties analysed in this dataset fall into three language types: Indigenised L2 varieties (L2), high-contact L1 varieties (L1c), low-contact L1 varieties (L1t) [24]. This categorisation has been adopted from eWAVE [24]. Precisely, English varieties which can be mapped onto existing eWAVE varieties are assigned the language type specified in eWAVE. eWAVE relied on expert linguists who categorised their varieties of expertise to one of the available language types. It goes without saying that the socio-historical and sociolinguistic background of the respective varieties was carefully considered in this process. English varieties which are not listed in eWAVE were likewise categorised based on their socio-historical background and status (e.g. standard L1 variety, traditional dialect). This applies to British English, Canadian English, Trinidadian English, English dialects in the Midlands, and Hebridean English.

In brief, indigenised L2 varieties comprise varieties mainly in former colonies of the British empire (e.g., Indian English) [24]. Although these varieties are labelled as non-native varieties in eWAVE most indigenised L2 varieties are (increasingly) spoken as native varieties and are often part of speakers’ multilingual repertoires. High-contact L1 varieties comprise English varieties which, in the course of their history, have been in contact with either other English varieties or other languages. This type includes varieties in former settlement colonies (e.g., New Zealand English), language-shift varieties such as Irish English, and standard L1 varieties (e.g., British English) [24]. Lastly, low-contact L1 varieties comprise regional non-standard varieties which have long since been established as mother tongue varieties and which have been comparatively isolated in their recent history [24].

Although the influence of geography on morphosyntax in English varieties is reported to be rather weak [34–36], region is included to control for potential geographic autocorrelation. The English varieties in this dataset are located in the following six geographic macro regions: Africa (Af), Americas (Am), Asia (As), the British Isles (BI), the Caribbean (Ca), and Oceania (Oc) [24,28].

### Syntheticity and grammaticity

Morphosyntactic complexity is operationalised as syntheticity (SI) and grammaticity (GI). These measures are well-established in corpus-based complexity research of English varieties [23,32,33] and are quantitative absolute measures, i.e., they are language-inherent and based on the number of explicit grammatical markers in the language system. To be more precise, syntheticity is defined as the number of bound grammatical markers in a corpus text, whereas grammaticity is defined as the number of all explicit grammatical markers, both bound and unbound, in a given corpus text. Bound markers include, for instance, nominal inflections like plural *-s*, or the adjectival comparative marker *-er*, verbal inflections like third-person singular *-s*, but also lexically conditioned allomorphs (e.g. past tense allomorphs such as *sang*, plural allomorphs such as *children*). Unbound markers comprise function words such as prepositions and conjunctions, auxiliary verbs, and modals (for a complete list, see https://github.com/Morphosyntactic-Variation-in-Englishes/syngram-complexity).

Syntheticity thus indexes the degree of inflectional marking in a given corpus text, and on an interpretational plane, in a given variety. Inflectional marking is a widely used complexity measure in typological studies [12,14,21]. Grammaticity indexes the overall degree to which a given corpus text, and hence variety, explicitly encodes grammatical meaning. In contrast to inflectional marking, grammaticity is only rarely employed in the typological literature (but see [21], for a similar measure).

On a technical note, the frequencies of the bound and unbound grammatical markers were automatically extracted based on their POS-tags from the POS-tagged corpus texts using a custom-made Python script. The script returns both individual marker frequencies, the total number of bound markers (SI), and the sum of all, i.e. bound and unbound, explicit grammatical markers (GI) per corpus text. In addition, the total number of tokens for each corpus text is also extracted.

### Statistical analysis

To test the effect of language contact as non-native acquisition on morphosyntactic complexity, I construct two theoretically motivated models. Specifically, for each operationalisation of morphosyntactic complexity, i.e. syntheticity and grammaticity, one model is constructed. Both models include morphosyntactic complexity as dependent variable and the independent variables *log*_10_ number of native speakers [12,15], proportion of non-native speakers [11,12,14], and the factor language type with three levels (traditional L1 (L1t), high-contact L1 (L1c), and indigenous L2 (L2)). The models further include a varying intercept for region to control for effects of geographic autocorrelation and a varying intercept for corpus to account for between-corpus differences. Decorrelated by-region varying slopes for the two demographic triggers are also included. Models with correlated slopes resulted in singular fit, hence, the slopes were decorrelated instead of completely removing them. The reason for including by-region varying slopes is that the effect of speaker numbers on complexity might vary according to region [14]. In the context of English varieties, a similar control seems justified as the effect of speaker-related triggers might have a stronger effect on varieties in certain regions. For instance, Asian varieties tend towards deletion while African varieties tend towards preservation [37]. In terms of syntheticity and grammaticity this means that Asian varieties might drop markers whereas African varieties might preserve them.

Additionally, a recent scientific exchange including the re-analyses of the same typological data [19,20] highlights the critical difference that inclusion or removal of slopes for speaker-related triggers may make. Against this background, I explicitly refrain from testing the significance of the varying slopes in order to remove or keep them in the model. Rather, I consider them a crucial part of the experimental set-up. As the corpus texts differ substantially in length, the logarithmically transformed total number of tokens in each corpus text is added as an exposure variable.

Technically, the models are implemented in R v4.5.2 [38] drawing on the lme4 library [39]. To be more precise, I fit generalised linear mixed effects models with a negative binomial distribution for overdispersed count data. When initially fitting the models with a Poisson distribution, the customarily used Pearson Chi-squared test indicated significant overdispersion (Table 2). None of the models exhibits zero-inflation or harmful multicollinearity.

**Table 2:** Dispersion ratio by model.

Model name	Dispersion	Estimated Chi-squared
SI_max_nocorr	6.84	$\chi_{\left(1918\right)}^{2}=13122$, p<0.001
SI_max_nocorr.nb	0.97	$\chi_{\left(1917\right)}^{2}=1862$, p=0.809
GI_max_nocorr	5.92	$\chi_{\left(1918\right)}^{2}=11346$, p<0.001
GI_max_nocorr.nb	0.98	$\chi_{\left(1917\right)}^{2}=1879$, p=0.73

## Results

According to the working hypothesis, larger numbers of native speakers and larger proportions of non-native speakers are expected to have a negative effect on syntheticity and grammaticity. In the same vein, higher levels of historic language contact, represented by the factor language type, i.e. the levels high-contact L1 and indigenous L2, should lead to a decrease in syntheticity and grammaticity, respectively. The statistical significance of fixed and random effects in both models was assessed by calculating likelihood ratio tests and profile confidence intervals. Note that the results of likelihood ratio tests are only reported if the reduced models converged without warnings.

### Syntheticity and language contact

**Fig 1:**
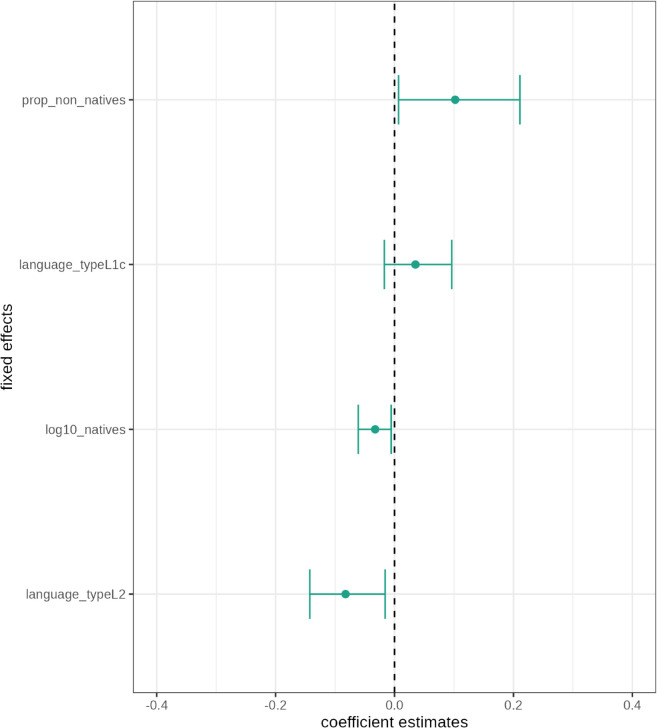
Dots-and-whiskers plot of fixed effects in the syntheticity model. Coefficients are on the log scale.

Fig 1 shows the profile confidence intervals of the fixed effects in the syntheticity model. The proportion of non-native speakers exhibits a positive effect on syntheticity. This effect might be considered marginally statistically significant. However, it needs to be stressed that the profile confidence interval for this effect is comparatively wide and indicates substantial estimation uncertainty. Nevertheless, the effect direction is theoretically interesting because it is unexpected and contrary to the hypothesis that larger proportions of non-native speakers lead to a decrease in bound markers. The number of native speakers has a significant negative effect on syntheticity ($\chi_{\left(1\right)}^{2}=5.027,p=0.025$). Thus, the effect of the number of native speakers is in line with the hypothesis that larger numbers of native speakers lead to a decrease in the number of bound markers. The number of native speakers thus affects syntheticity as initially expected. The theoretical construct language type, precisely, the level indigenous L2 varieties seems to have a significant negative effect on syntheticity. In contrast, the level high-contact L1 varieties has no significant effect on syntheticity. This is indicated by the inclusion of zero in the confidence interval of this effect. Overall, it needs to be noted that the coefficient estimates for all fixed effects are relatively small.

**Fig 2:**
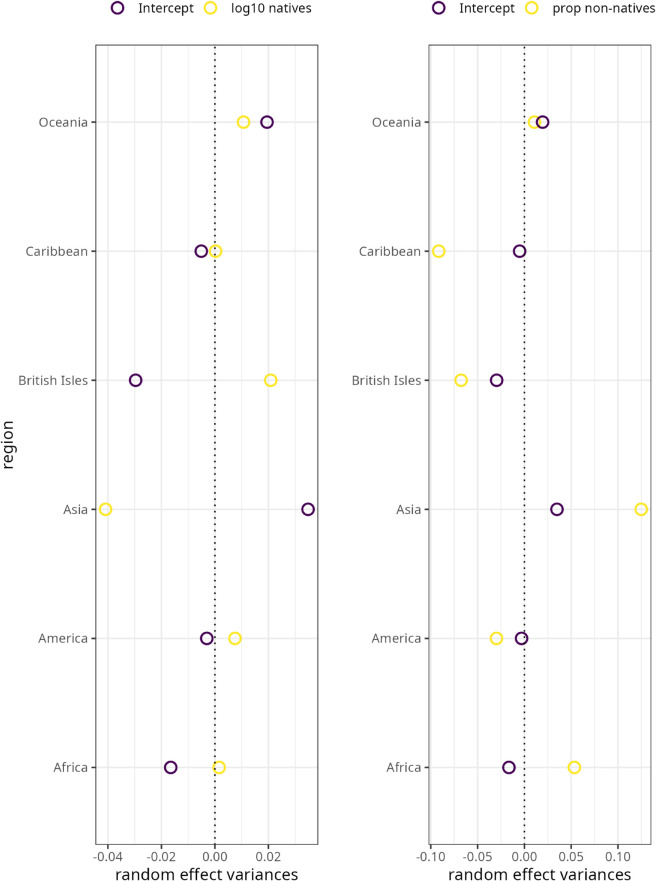
Random effects variances for region in the syntheticity model. (A) By-region varying intercept and slope variances for log10 number of native speakers. (B) By-region varying intercept and slope variances for proportion of non-native speakers. Coefficients are on the log scale.

Table 3 lists the confidence intervals of the varying intercepts for region and corpus. They both include zero which means that neither the effect of region ($\chi_{\left(1\right)}^{2}=0.337,p=0.562$) nor the effect of corpus are statistically significant. In fact, the inspection of the random effect variances for the two demographic triggers reveals that they are very close to zero (see Fig 2). Put differently, regional differences and differences due to corpus source do not account for the observed variation in syntheticity.

**Table 3:** Confidence intervals for varying intercepts.

Varying intercept	Low	High
Region	0	0.24
Corpus	0	0.12

### Grammaticity and language contact

**Fig 3:**
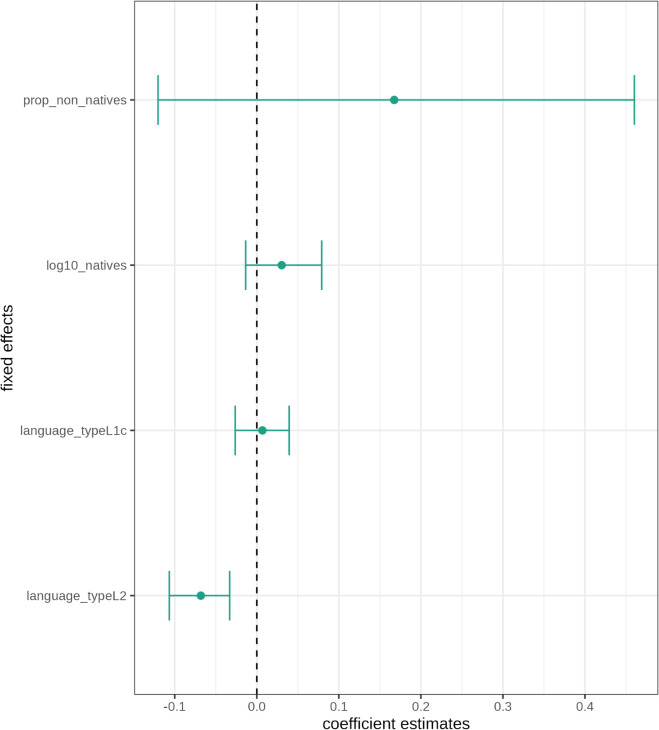
Dots-and-whiskers plot of fixed effects in the grammaticity model. Coefficients are on the log scale.

As visualised in Fig 3, none of the speaker related triggers exhibit a statistically significant effect. This means that neither the number of native speakers nor the proportion of non-native speakers influences grammaticity in the analysed English varieties when controlling for region and corpus. However, it needs to be noted that the effect directions are positive and therefore theoretically and linguistically relevant: Larger numbers of native speakers and larger proportions of non-native speakers are associated with higher grammaticity instead of lower grammaticity.

Language type at the level of indigenous L2 varieties emerges as the only significant predictor for grammaticity ($LRT=134.255,p(\chi^{2})<0$). Moreover, its effect is also negative and therefore in line with the working hypothesis that larger degrees of contact lead to less grammaticity. The effect for the level high-contact L1 varieties is non-significant.

**Fig 4:**
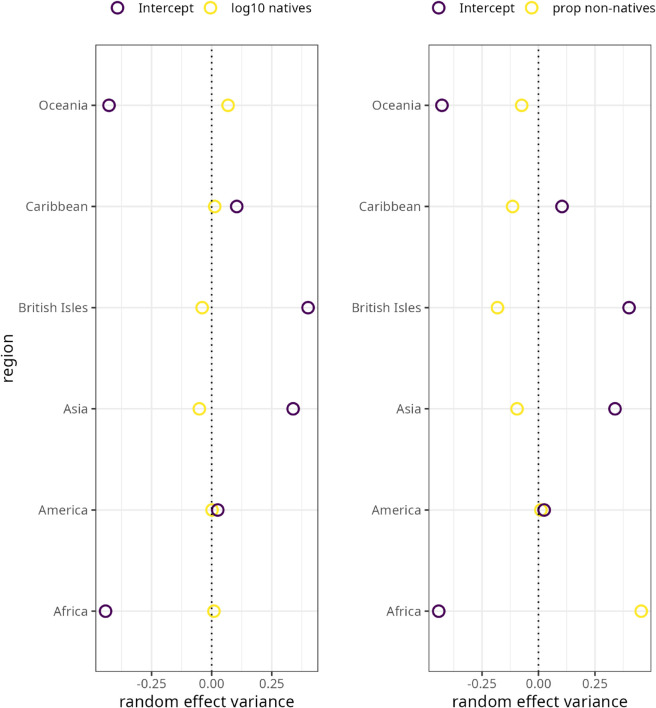
Random effects variances for region in the grammaticity model. (A) By-region varying intercept and slope variances for log10 number of native speakers. (B) By-region varying intercept and varying slope variances for proportion of non-native speakers. Coefficients are on the log scale.

Fig 4 presents the random effects variances of the demographic triggers for region. The varying intercept for region is highly statistically significant ($\chi_{\left(1\right)}^{2}=27.591,p<0$) whereas the varying intercept for corpus ($\chi_{\left(1\right)}^{2}=0.7679,p=0.381$) is not. In other words, variation in grammaticity is mainly explained by the varying intercept for region.

## The influence of language contact on morphosyntactic complexity in English varieties

This paper tested the influence of language contact as non-native acquisition on syntheticity and grammaticity in corpora of English varieties. Language contact was operationalised as the number of native speakers and the proportion of non-native speakers, which often feature as key explanatory triggers for variation in language complexity [11–13,15,30]. Language type, a theoretical construct which approximates historic language contact scenarios, was also included. The paper thus illuminates a hotly debated issue in the sociolinguistic typological research community from the usage-based perspective of intra-language variation.

According to contact scenarios described in the literature [16] and an influential empirical study [15], all predictors were expected to have a negative effect on syntheticity and grammaticity conditioned on the random structure. However, this initial assumption is only partially borne out by the data. I find that syntheticity is indeed influenced by language contact to some extent, i.e. the number of native speakers has a significant negative effect on syntheticity. Specifically, varieties with more native speakers exhibit less bound markers than varieties with less native speakers. The proportion of non-native speakers shows a positive, marginally significant effect on syntheticity. However, this effect is marked by substantial estimation uncertainty. That said, the effect direction is theoretically interesting and noteworthy as larger proportions of non-native speakers are associated with more bound markers. In contrast, both speaker-related triggers show no significant effect on grammaticity. Language type has a negative significant effect on both syntheticity and grammaticity. Hence, the level indigenous L2 varieties, which constitutes the largest degree of historic language contact, is associated with less syntheticity and grammaticity. Interestingly, the varying intercept for geographic region is non-significant in the syntheticity model but highly significant in the model with grammaticity. Corpus does not seem to influence morphosyntactic complexity in either model despite assumed corpus heterogeneity.

Overall, it is fair to say that these results are a reflection of the previous, controversial and inconclusive findings in the typological literature. This, in itself, is an important finding. For one, the results from intra-language variation in English corroborate the cross-linguistic typological findings – even if this means that the question on the influence of speaker numbers on complexity cannot be conclusively answered. Regardless, the paper does provide some evidence for the influence of *historic* language contact on linguistic structure. Further, these results are important because next to nothing is known about the relationship between sociodemographic triggers and intra-language complexity. Consequently, the paper is one of the first to shed light on this relationship. The present findings also raise questions of far-reaching theoretical implications beyond the English-varieties context.

The negative effect of the number of native speakers on syntheticity is theoretically expected and in line with the idea that languages spoken by larger native speaker communities are also used for wider communication outside these speaker communities [15]. This, in turn, leads to a decrease in complexity over time. The first question, then, is why grammaticity (i.e. the number of bound and unbound markers counted together) is not influenced by the number of native speakers in the same way.

The shifting response of morphosyntactic complexity to sociodemographic triggers, especially the number of native speakers, is well-known and has led to debates on contact and complexity [11,19,20]. Although some studies [15,40] find a negative correlation between the number of native speakers and various operationalisations of complexity, a growing body of large-scale typological studies casts doubt on these earlier findings. Some studies even report (moderate to weak) positive effects of the number of native speakers on complexity [11,21]. The study by [21] is particularly relevant here because one of their complexity measures, informativity, is basically the typological equivalent of grammaticity in English varieties. In any case, they found that the number of native speakers and the proportion of non-native speakers both had a weak positive effect on informativity which parallels my findings in regard to grammaticity (albeit the effects in my paper are not significant). One possible explanation for the contrary findings is that, indeed, some grammatical phenomena are more responsive to extra-linguistic triggers than others [12], i.e. it matters how complexity is measured and operationalised. [12] report that the number of native speakers has a significant negative effect on verbal inflectional synthesis but not on grammatical gender. This explanation is further supported by very recent evidence from language-internal complexity variation in English varieties [41]. Using an information-theoretic measure of complexity, no significant correlations between the number of native speakers or the proportion of non-native speakers was found. This study is highly comparable to the present paper because it is based on the same database but uses a different measure of complexity and a slightly different set of predictor variables. Thus, the two studies together provide truly incremental and robust evidence in the sense of [42] for the fact that different complexity measures respond more – or less – to speaker-related and other extra-linguistic triggers. Nevertheless, different data types (e.g. atlas data) and more complexity measures of the same varieties should be investigated to bolster these findings. Another (or additional) explanation is, of course, that some morphosyntactic features may be more susceptible to language internal (rather than external) pressures or the interaction of language internal and external pressures as suggested by [43].

Assuming that different morphosyntactic features are indeed distinctly responsive to sociodemographic triggers, the diverging findings on syntheticity and grammaticity warrant further discussion. The negative effect on syntheticity suggests that contact-induced simplification may preferentially affect bound grammatical marking rather than unbound grammatical marking. In fact, unbound grammatical marking, generally referred to as analytic marking, is often associated with transparency [32,44,45]. This implies that unbound marking by virtue of being more transparent might be easier to learn than bound marking. Unbound marking might thus also be more faithfully transmitted in contact situations. This is also in line with a pertinent study on adult learner language: Adult learners typically rely on lexical rather than grammatical marking for conveying information [46]. Hence, syntheticity, which is presumably harder to learn and transmit, is more affected by language contact, whereas grammaticity is not. Incidentally, some of the typological studies which do find negative effects of language contact on linguistic structure analyse bound grammatical marking [12,14,15,47]. Furthermore, [15] explicitly report that languages spoken by larger numbers of native speakers comprise more unbound markers and generally prefer lexical or analytic strategies to encode grammatical meaning. Similarly, [47] differentiate between morphological and syntactic features and find that morphological (bound) marking is inversely correlated with exotericity, i.e. language contact. In other words, more language contact tends to be associated with less bound marking.

The second question is, why the proportion of non-native speakers has a positive, rather than negative, effect on syntheticity. This is of theoretical and linguistic relevance albeit the effect is at best marginally significant. Arguably, the proportion of non-native speakers is a more direct measure of language contact as non-native acquisition than the number of native speakers [12]. Yet, in most of the typological literature, the proportion of non-native speakers [12,14] or approximations thereof [11] perform worse as predictor for complexity or, like in this study, have a positive instead of a negative effect. A reason for this theoretically unexpected behaviour, which so far has not been sufficiently considered, is the conceptualisation of non-native speaker proportions. Maybe, the proportion of non-native speakers does not really approximate a contact scenario with large-scale (adult) second/non-native language acquisition but rather one of long-term language contact with child bilingualism. In this case, the positive effect direction of the proportion of non-native speakers would make sense and be in line with the literature [16,18]. In the case of this paper, the sources of the proportion of non-native speakers are well documented so that the (reported) proficiency of the non-native speakers is known. Namely, non-native speakers here mostly refers to speakers who report to speak English well, very well or not well [28]. Thus, this count can also include bilingual speakers who did not report English as their main or dominant language at home (not many census surveys account for multilingualism). As suggested elsewhere [48] more fine-grained non-native speaker data with different proficiency levels is required to shed light on this issue. On a related note, present-day proportions of non-native speakers might simply not reflect the proportions of the original historic contact situations which gave rise to present-day languages and varieties. The concern of time depth has also been raised and discussed by [14]. In contrast to the proportion of non-native speakers, the number of native speakers might be a better predictor (for some complexity measures like syntheticity) because the size of present-day native speaker populations can be seen as the result of historic contact situations and concomitant spread of the languages/varieties in the past. Time depth also explains the comparatively stable and theoretically expected effect of language type on both syntheticity and grammaticity. I included this theoretical construct in the analysis exactly because it is a proxy for historic language contact.

The third question relates to the use of speaker numbers *per se*. The results obtained in this paper raise the very general but important question of whether speaker numbers are an adequate predictor for language complexity at all because their effect and its direction is highly unstable. The reliability of available (non-)native speaker numbers is mostly glossed over and should be considered in quantitative typological studies making strong claims about the effect of language contact [48]. As [49, 4] notes in regard to speaker numbers for English varieties: “The statistics of English worldwide is an inexact science, and utilises information of varying reliability”. The same is certainly true for any type of speaker statistics of the world’s languages.

Finally, geographic region only emerged as significant in the grammaticity model. This is surprising because recent studies report highly signifiant effects of geography (and genealogy). In fact, when controlling for geographic region (and genealogy) effects of speaker data or other demographic triggers are either weak or non-significant [20,21,50]. Hence, effects of speaker-related triggers only emerge when geography does not matter as in the syntheticity model.

Apart from these theoretical contributions, the paper also makes two methodological contributions. First, it showcases how cross-linguistic typological research questions can be addressed from the well-charted landscape of intra-language variation in English [32]. Second, my findings provide usage-based evidence from naturalistic spoken materials to a discourse that is dominated by evidence from descriptive atlas materials. Thus, the paper exemplifies how language structure is shaped by language usage [51].

Taken together, I hedge that the above points relating to speaker numbers may at least partially explain the contradictory findings reported in regard to the influence of language contact on complexity. Nevertheless, the crucial question of whether language contact, operationalised as non-native acquisition and native speaker population affect language complexity - or not - remains unresolved.
